# MicroRNA-99b predicts clinical outcome of osteosarcoma and suppresses tumor cell proliferation, migration and invasion

**DOI:** 10.1186/s13000-019-0889-y

**Published:** 2019-10-24

**Authors:** Xin Shi, Xingfa Guan

**Affiliations:** Department of Orthopaedics, Qilu Hospital Huantai Branch, No.2198, Huantai Road, Zibo, 256400 Shandong China

**Keywords:** Osteosarcoma, miR-99b, Prognosis, Biological function, Tumor progression

## Abstract

**Background:**

Osteosarcoma (OS) is a malignancy predominantly occurred in children and adolescents. Numerous microRNAs are involved in the pathogenesis of various cancers. This study aimed to investigate the expression profiles of miR-99b and its prognostic value in OS patients, and further analyze the biological function of miR-99b in the tumor progression by using OS cells.

**Methods:**

Expression of miR-99b was measured using quantitative real-time PCR. Kaplan-Meier survival curves and Cox regression analysis were performed to evaluate the prognostic value of miR-99b. OS cell lines were used to investigate the effects of miR-99b on cell proliferation, migration and invasion.

**Results:**

A significant decreased expression of miR-99b was observed in the OS tissues and cell lines respectively compared with the normal tissues and cells. Aberrant expression of miR-99b was associated with the patients’ metastasis and TNM stage, and could be used to predict the prognosis of OS. The expression of miR-99b was regulated in vitro by cell transfection, and we found that the overexpression of miR-99b led to suppressed cell proliferation, migration and invasion, whereas the knockdown of miR-99b resulted in the opposite results.

**Conclusions:**

In one word, the aberrantly expressed miR-99b serves a prognostic biomarker for OS patients. OS cell proliferation, migration and invasion can be inhibited by the overexpression of miR-99b, suggesting that the methods to increase miR-99b expression may be novel therapeutic strategies in OS.

## Introduction

Osteosarcoma (OS) is a primary bone tumor and characterized by the bimodal distribution that mainly occurs in children and adolescents [1]. Statistics indicate that the incidence of OS is approximately 4.4 per 100,000, and this malignancy ranks the second common primary tumor in bone [2]. Some risk factors have been identified to be closely related with the occurrence of OS, including medical history of radiation or chemotherapy, benign bone lesions and some genetic conditions [3]. Surgical operation is the predominant chose for OS treatment, and the adjuvant therapy, such as chemotherapy, contributes to the reduction of mortality [4]. Currently, the 5-year survival rate of this malignancy has reached to 80% [5]. However, the prognosis remains dismal for the OS patients diagnosed with advanced tumors [6]. Thus, accurate prognosis and efficient therapy are urgently needed to improve the treatment of OS.

Targeted cancer therapy has received increasing attention in the cancer research field as a large number of deregulated key molecules have been identified in various tumors [7, 8]. Numerous microRNAs (miRNAs) with abnormal expression patterns have been detected in human cancer samples, and they are considered to be involved in the pathogenesis of various cancers [9, 10]. MiRNAs are a groups of small noncoding RNAs with the important regulatory function in the gene expression at post-transcriptional levels [11]. In addition, miRNAs serve critical roles in the regulation of some cell processes, such as cell proliferation, migration, invasion, differentiation and cell apoptosis [12]. In OS, some aberrant miRNAs have also been identified, such as miR-124 [13] and miR-491 [14], with close correlation with diagnosis, prognosis and tumorigenesis. Decreased expression of microRNA-99b (miR-99b) has been reported in several types of human cancer, including gastric cancer [15] and colorectal carcinoma [16]. The regulatory role of miR-99b in the chemotherapy of OS has been analyzed in a study by Gougelet et al. [17], but its expression pattern and clinical significance in OS remain elusive.

In this study, the expression of miR-99b in the patients with OS was investigated, and the potential clinical significance of miR-99b was evaluated. To further understand the functional role of miR-99b in OS progression, its effects on tumor cell processes were assessed.

## Materials and methods

### Patients and tissue collection

OS tissues and paired adjacent normal tissues were collected from 110 patients who had been subjected to resection surgery in Qilu Hospital Huantai Branch from June 2010 to May 2013. The patients included 59 males and 51 females with an average age of 20.98 ± 5.08 years old (age range of 9–29 years old). None of patients had received any therapy prior to the surgery, and all the patients were pathologically diagnosed as OS after the surgery. The collected tissues were graded into IA -IIA stage (*n* = 38) and IIB - III stage (*n* = 72) according to the Enneking - Musculoskeletal Tumour Staging System. All the tissues were frozen in liquid nitrogen and stored at − 80 °C for further use. The demographic and clinicopathological characteristics and the survival information in a 5-year follow-up survey (range of 0–60 months) were recorded for the subsequent analyses. The cases died from other events were excluded from this study. A signed written informed consent was obtained from each patient and the experimental procedures were in accordance with the guideline of the Ethics Committee of Qilu Hospital Huantai Branch.

### Cell culture

Four OS cell lines (HOS, MG63, SaOS2 and U2OS) and one normal human osteoblast cell line (hFOB1.19) were obtained from the Shanghai Cell Bank of Chinese Academy of Science (Shanghai, China). The cells were maintained in DMEM medium (Gibco, CA, USA) supplemented with 10% FBS (Gibco, CA, USA) and cultured at 37 °C with 5% CO_2_.

### Cell transfection

To regulate the expression of miR-99b in OS cells, miR-99b mimic (5′-CACCCGUAGAACCGACCUUGCG-3′), miR-99b inhibitor (5′-CGCAAGGUCGGUUCUACGGGUG-3′) and the negative control sequence (miR-NC, 5′-UUCUCCGAACGUGUCACGUTT-3′) were synthesized in Ribobio (Guangzhou, China). The vectors above were separately transfected into the OS cells using Lipofectamine 2000 (Invitrogen, Carlsbad, CA, USA) following the manufacturers’ protocols.

### RNA extraction and quantitative real-time polymerase chain reaction (qRT-PCR)

Total RNA was extracted from the tissues and cells using the TRIzol reagent (Invitrogen, Carlsbad, CA, USA) as per the standard method. The synthesis of cDNA from the RNA was performed using a PrimeScript RT reagent kit (TaKaRa, Shiga, Japan) as per the manufacturers’ instruction. The expression of miR-99b was measured by qRT-PCR, which was carried out using a SYBR green I Master Mix kit (Invitrogen, Carlsbad, CA, USA) on a 7500 Real-Time PCR System (Applied Biosystems, USA). U6 was used as an endogenous control, and the final expression value was calculated using the 2^−ΔΔCt^ method. The primers used for this analysis were as follows: miR-99b F: 5′-GCCGAGCACCCGTAGAACCG-3′, R: 5′-CTCAACTGGTGTCGTGGA-3′; U6 F: 5′-CTCGCTTCGGCAGCACA-3′, R: 5′-AACGCTTCACGAATTTGCGT-3′.

### Cell proliferation analysis

After 48 h of cell transfection, the tumor cells were seeded in 96-well plates to examine the ability of proliferation using the MTT method. The cell plates were maintained in an incubator at 37 °C for 3 days, and 10 μL MTT (5 mg/mL) was added at the time points of 0, 24, 48 and 72 h followed by further 4 h incubation. Then a volume of 150 μL DMSO was added in the wells. After the incubation, a microplate reader was adopted to measure the absorbance of the cells at 490 nm.

### Cell migration and invasion analysis

Transwell chambers (Corning, USA) with Matrigel precoating (for invasion assay) or without Matrigel coating (for migration assay) were used to analyze the migration and invasion abilities of the OS cells. Tumor cells with serum-free culture medium were seeded into the upper chambers, and the lower chambers included medium contained 10% FBS. After 48 h incubation at 37 °C, the cells in the lower chambers were stained and the cell number in five random fields was counted under an inverted microscope (Olympus Corporation, Tokyo, Japan).

### Statistical analysis

SPSS 21.0 software (IBM, Chicago, IL) and GraphPad Prism 7.0 software (GraphPad Software, Inc., USA) were used for data analyses. All the experiments and examinations were performed at least three times. Data were expressed as mean ± SD. Student’s t test and one-way ANOVA were used to assess the between-group differences. Relationship between miR-99b and clinical features was analyzed using Chi-square test. The Kaplan-Meier (KM) method was used to plot survival curves for the OS patients, and Cox regression analysis was applied to evaluate the prognostic value of miR-99b. *P* < 0.05 was considered statistically significant.

## Results

### Decreased expression of miR-99b in OS

**Fig. 1:**
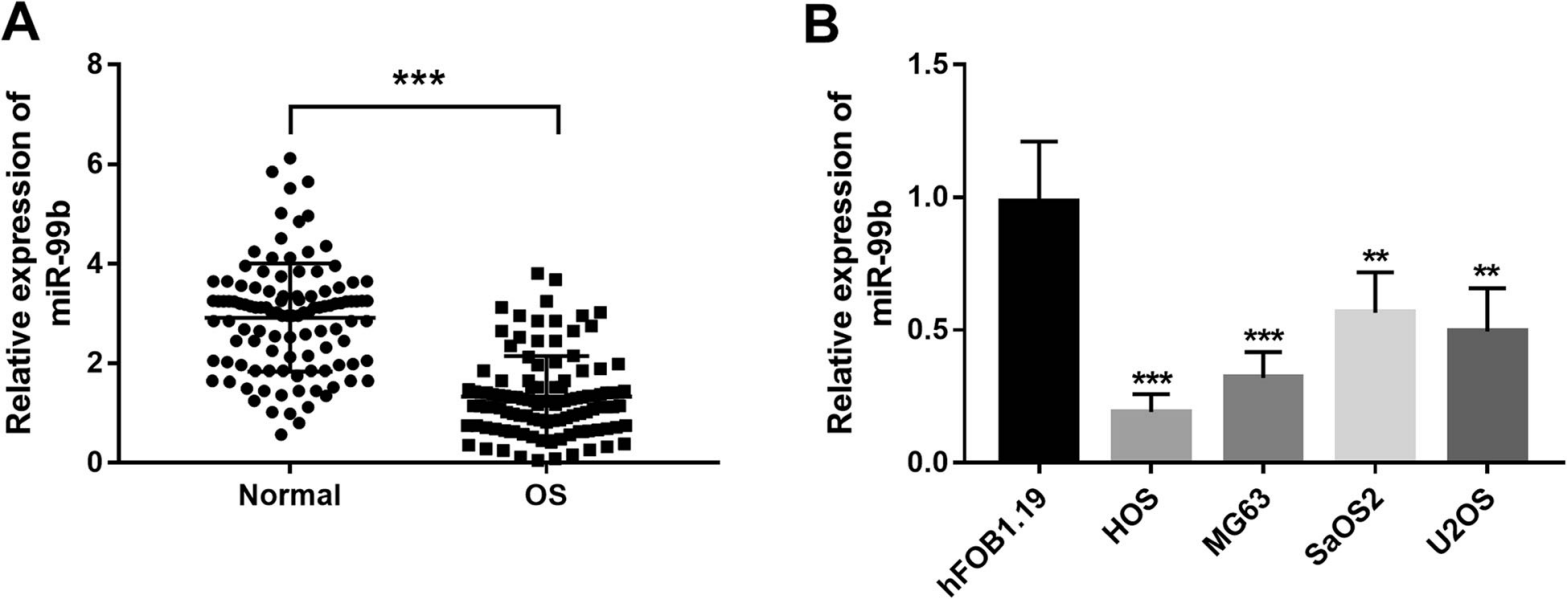
Expression of miR-99b examined by qRT-PCR in OS. **a** Expression of miR-99b was decreased in OS tissues compared with the normal tissues. **b** Expression of miR-99b was downregulated in the four OS cell lines compared with the normal cells. **P < 0.01, **P < 0.001

According to the qRT-PCR, we found that the expression of miR-99b in the tumor tissues was obviously downregulated compared with the normal tissues (1.34 ± 0.80 vs. 2.91 ± 1.09, *P* < 0.001, Fig. 1a). In addition, the expression patterns of miR-99b were further confirmed in the OS cells, which also performed a significantly lower expression than the normal cells (all *P* < 0.01, Fig. 1b).

### Association between miR-99b and the clinicopathological characteristics of the patients

All the demographic and clinical features were summarized in Table 1. By using the median value (1.245) of miR-99b expression, the OS patients were grouped into low (*n* = 56) and high (*n* = 54) miR-99b expression groups. From the data of Chi-square test, we observed that the expression of miR-99b was associated with metastasis (*P* = 0.008) and TNM stage (*P* = 0.003). However, no relationship was found between miR-99b and age, gender, tumor size or differentiation (all *P* > 0.05).

**Table 1:** Association of miR-99b and the clinical characteristics of the OS patients

Features	Total No. *N* = 110	miR-99b expression		*P* values
		Low (*n* = 56)	High (*n* = 54)	
Age (Years)				0.724
≤ 20	45	22	23	
> 20	65	34	31	
Gender				0.989
Female	51	26	25	
Male	59	30	29	
Tumor size (cm)				0.311
≤ 8	66	31	35	
> 8	44	25	19	
Distant metastasis				0.008
Negative	55	21	34	
Positive	55	35	20	
Differentiation				0.876
Well/moderate	67	34	33	
Poor	43	22	21	
Clinical stage				0.003
IA - IIA	38	12	26	
IIB - III	72	44	28	

### Prognostic significance of miR-99b in the patients with OS

**Fig. 2:**
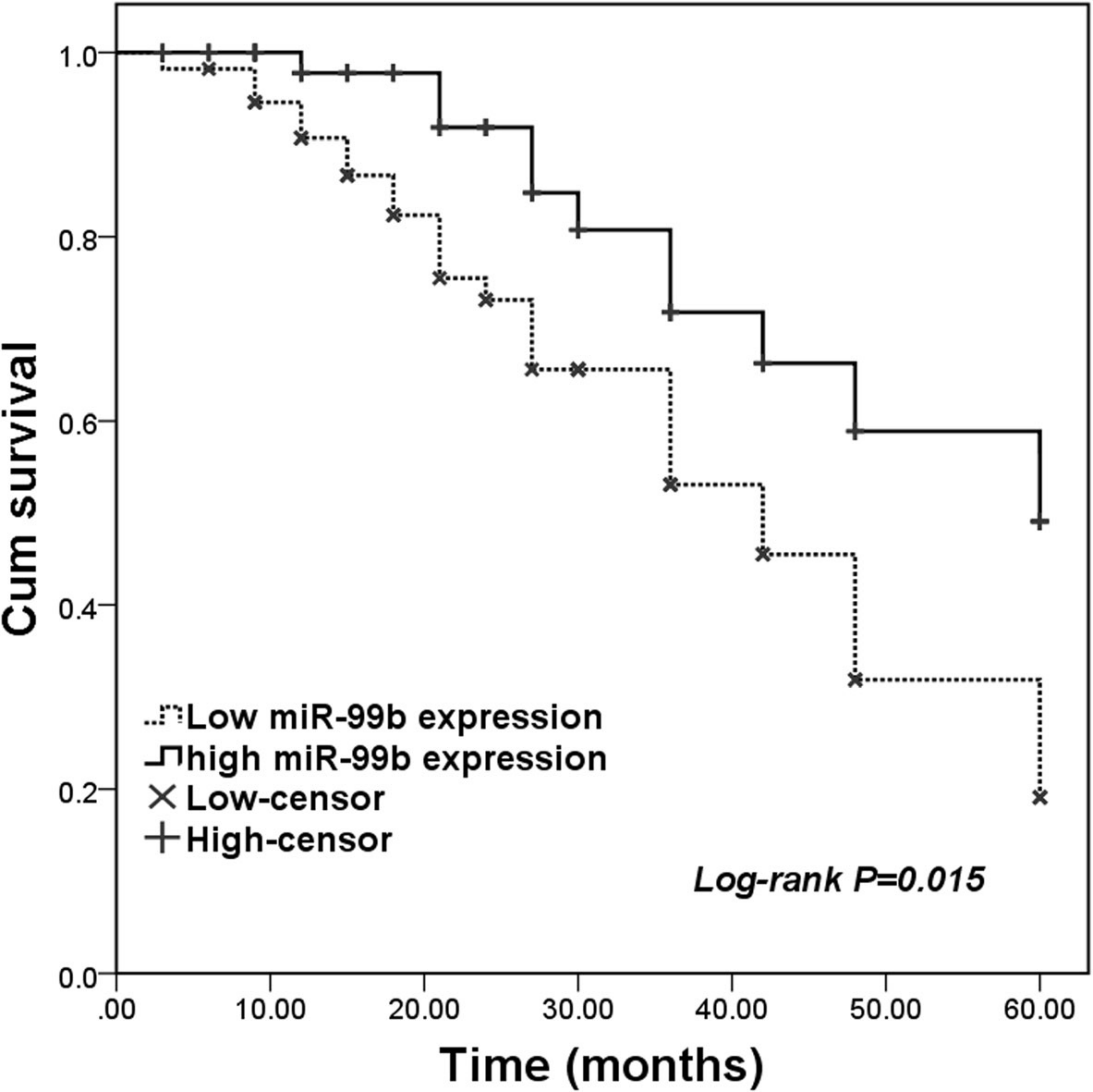
Kaplan-Meier survival curves in the patients with OS. Patients with low expression of miR-99b had shorter survival time than those with high miR-99b expression. Log-rank *P* = 0.015

Given the dysregulation in the expression of miR-99b in OS clinical samples, this study further investigated the clinical significance of miR-99b in the prognosis of OS. As shown in Fig. 2, the patients with low levels of miR-99b had markedly shorter survival time compared with those with high levels of miR-99b (log-rank *P* = 0.015). According to the 60 months follow-up survey, the OS patients had a median survival time of 48 months, and this value in the cases with low miR-99b was 42 months. To confirm whether the relationship between miR-99b and the overall survival was independent, a Cox regression analysis was performed. The univariate analysis results revealed that the expression of miR-99b, distant metastasis, differentiation and clinical stage were all associated with the patients’ overall survival (all *P* < 0.05, Table 2). The further multiple assay data indicated that miR-99b expression was an independent prognostic indicator for the overall survival in the patients with OS (HR = 2.897, 95% CI = 1.231–4.628, *P* = 0.019).

**Table 2:** Cox regression analysis in the patients with OS

Variables	Univariate analysis			Multivariate analysis		
	HR	95% CI	*P* value	HR	95% CI	*P* value
miR-99b	3.012	1.622–6.185	0.009	2.897	1.231–4.628	0.019
Low vs. High						
Age (years)	0.867	0.456–1.649	0.664	0.937	0.482–1.822	0.848
> 20 vs. ≤ 20						
Gender	1.258	0.650–2.435	0.496	1.117	0.552–2.258	0.758
Male vs. Female						
Tumor size (cm)	0.940	0.490–1.803	0.853	1.022	0.517–2.020	0.950
> 8 vs. ≤ 8						
Distant metastasis	2.018	1.211–3.185	0.033	1.279	0.631–2.595	0.495
Positive vs. Negative						
Differentiation	1.912	1.035–2.585	0.043	1.322	0.668–2.614	0.423
Poor vs. Well/moderate						
Clinical stage	1.969	1.104–2.613	0.038	1.758	0.755–4.093	0.191
IIB - III vs. IA - IIA						

### Inhibitory effects of miR-99b on cell proliferation, migration and invasion of OS cells

**Fig. 3:**
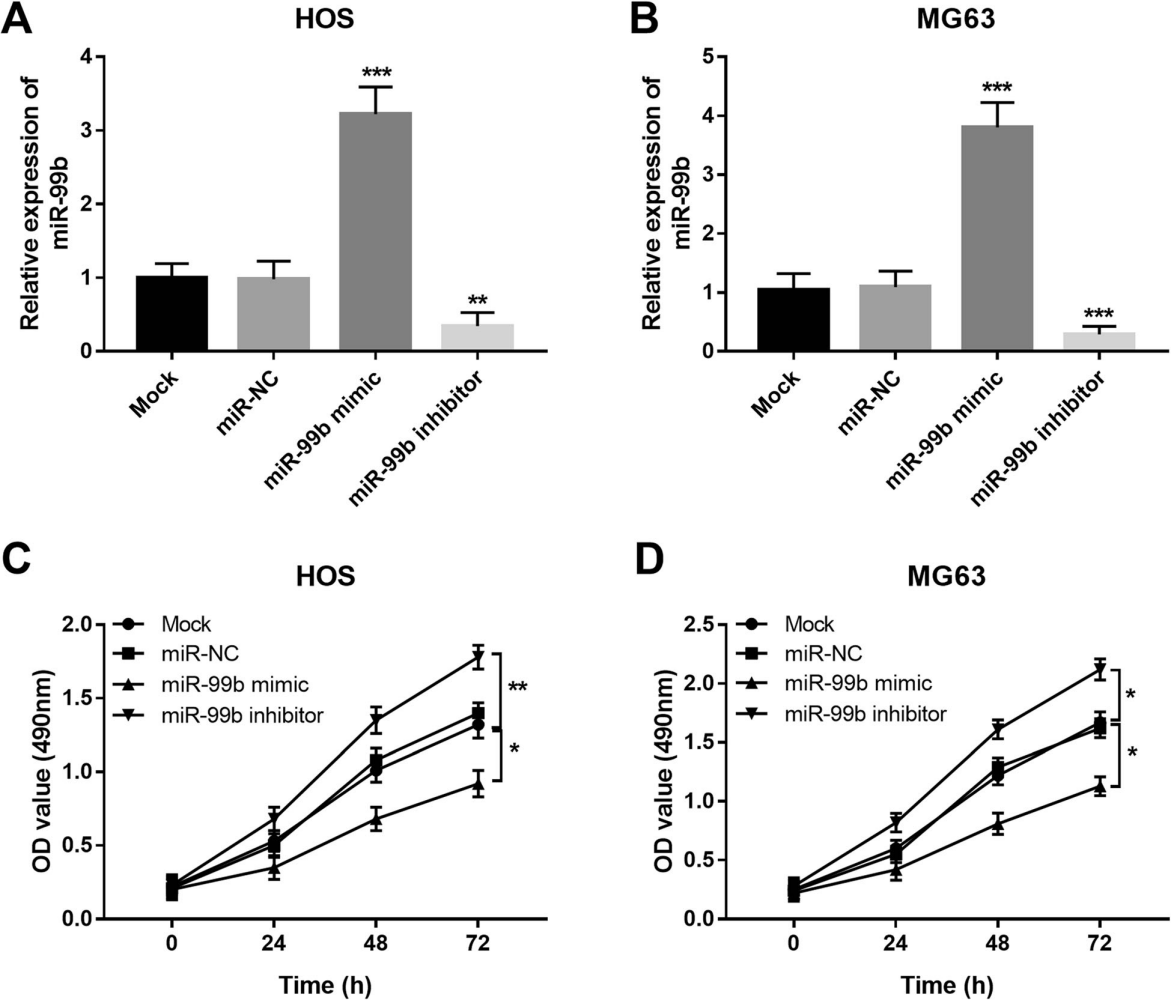
Effects of miR-99b on cell proliferation of HOS and MG63 cells. **a** and **b** Expression of miR-99b was upregulated by the miR-99b mimic, but was downregulated by the miR-99b inhibitor. **c** and **d** Tumor cell proliferation was promote by the downregulation of miR-99b, while was inhibited by the upregulation of miR-99b. *P < 0.05, **P < 0.01, **P < 0.001

To further understand the function of miR-99b in OS progression, HOS and MG63 were applied since they had dramatically low expression of miR-99b. By cell transfection, the expression of miR-99b in both the two cell lines was successfully upregulated by the miR-99b mimic, while was downregulated by the miR-99b inhibitor (all *P* < 0.01, Fig. 3a and b). With the use of MTT assay, the cell proliferation of the two cell lines was enhanced by the knockdown of miR-99b, but was inhibited by the overexpression of miR-99b (all *P* < 0.05, Fig. 3c and d).

**Fig. 4:**
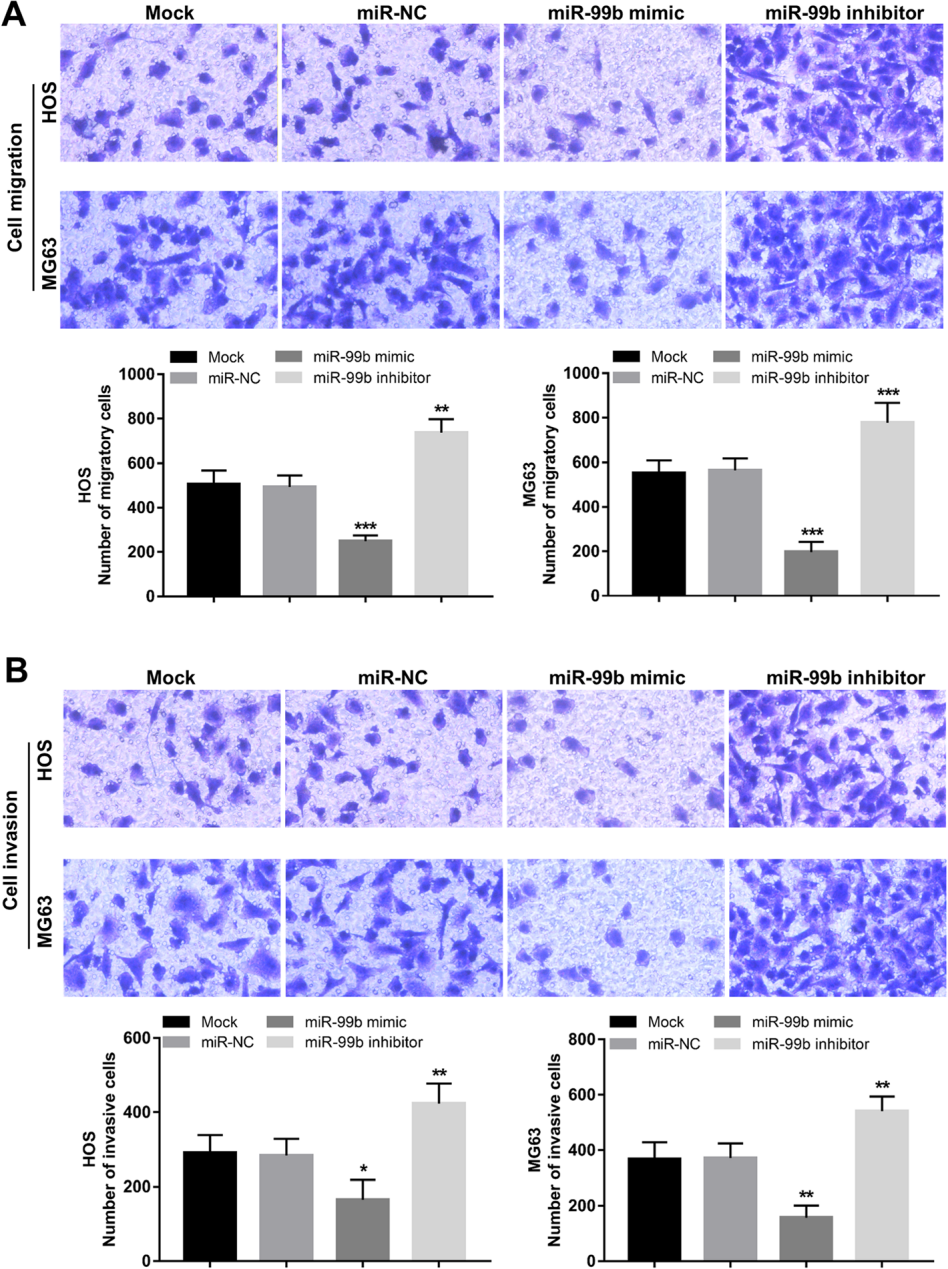
Effects of miR-99b on cell migration and invasion of HOS and MG63 cells. The overexpression of miR-99b in HOS and MG63 could suppress the cell migration (**a**) and invasion (**b**), but the reduction of miR-99b led to the opposite results. *P < 0.05, **P < 0.01, **P < 0.001

The abilities of migration and invasion were measured in the HOS and MG63 cells after cell transfection by using transwell chambers. As shown in Fig. 4a, the overexpression of miR-99b could suppress, whereas the reduction of miR-99b could promote the cell migration (all *P* < 0.01). For the invasion assay, we found that the tumor cell invasion was promoted by the knockdown of miR-99b, but was suppressed by the overexpression of miR-99b (all *P* < 0.05, Fig. 4b).

## Discussion

Accumulated studies indicate that tumor progression involves the dysregulation of a large number of molecules, of which miRNAs are an important component [18]. Numerous ectopic miRNAs have been identified in various types of tumor and serve critical roles in the pathogenesis of malignancies [19]. For example, the expression of miR-144 was reported to be downregulated in gastric cancer cell lines compared with the normal cells, and the overexpression of miR-144 in tumor cells resulted in inhibited cell proliferation and invasion [20]. In NSCLC tissues, miR-34b was downregulated and associated with the degree of differentiation and pathological stage of the patients, indicating its potential to serve as a biomarker for this malignancy [21]. The aforementioned studies suggest the closely relationship between miRNAs and human malignancies. In OS, there are also some miRNAs with aberrant expression profiles. A study by Chu et al. shown that the expression of miR-136 was reduced in OS tissues and cells, and might be involved in the regulation of tumor cell proliferation, migration and invasion [22]. Another study by Xia et al. found that miR-377 served a tumor suppressor in the tumor progression, which evidenced by the inhibited effects of miR-377 on tumor growth and the enhanced effect on cell apoptosis [23]. Similarly, the decreased miR-139 in OS was also been identified as a suppressor in tumorigenesis, and its suppressive effects on tumor cell proliferation and invasion were exerted by targeting ROCK1 [24]. Taken together, it is important to identify more deregulated miRNAs in the development of OS.

In this study, we found a significantly decreased expression of miR-99b in both the tissue and cell samples of OS when compared to the corresponding normal controls. Thus, we considered that the aberrant miR-99b might play a tumor suppressor role in OS. The downregulated expression patterns of miR-99b have been determined in other human cancers, and it has been demonstrated to serve as a tumor suppressor. For instance, miR-99b expression was demonstrated to be reduced in gastric cancer tissues and cell lines, and it could suppress the tumor cell proliferation and cell cycle by targeting IGF-1R [15]. In colorectal carcinoma, the expression of miR-99b was also downregulated in tumor tissues, and tumor cell migration could be inhibited by the overexpression of miR-99b [16]. In our research cohort, we further investigate the relationship between miR-99b expression and the clinical features of the patients, and found that the decreased expression of miR-99b was associated with metastasis and TNM stage. Thus, we considered that the deregulated expression of miR-99b might be related with the development of OS.

Emerging studies highlight the clinical significance of miRNAs in the diagnosis and prognosis of human cancers [25]. The prognostic value of miR-99b has been analyzed in some cancers, such as clear cell renal cell carcinoma [26]. With the recorded survival information, we plotted the survival curves, which indicated that low miR-99b expression was related with shorter survival time. Moreover, the Cox regression data implied that the expression of miR-99b was an independent prognostic indicator. Thus, we considered that the decreased expression of miR-99b could predict poor prognosis of OS.

Considering the reduced expression of miR-99b in OS tissues and cell lines, its functional role in tumor progression was explored after the in vitro regulation of miR-99b. This study found that the OS cell proliferation, migration and invasion were all promoted by the knockdown of miR-99b, while were inhibited by the overexpression of miR-99b, which confirmed that miR-99b served a tumor suppressor in the progression of OS. Although we provided evidence for the inhibiting effects of miR-99b on OS progression, the underlying mechanisms remain unclear. Li and his colleagues have reported that miR-99b could suppress cervical cancer cell proliferation, invasion, migration and cell cycle by the inhibition of the PI3K/AKT/mTOR signaling pathway [27]. Liu et al. have demonstrated that the non-small cell lung cancer cell proliferation, migration and invasion abilities were downregulated by miR-99b through targeting Frizzled-8 (FZD8) [28]. Furthermore, FZD8 has been reported to serve as an oncogene in OS [29]. Therefore, we suspected that the effects of miR-99b in OS might exerted also through targeting FZD8 or via the PI3K/AKT signaling. Further studies need to be carried out to confirm our hypothesis on the molecular mechanisms. In addition to the limited understanding about the underlying mechanisms, another limitation of this study was the small sample size that might lead to limited accuracy of our results. Thus, future studies are needed with a large research cohort.

In conclusion, all the data in this study demonstrated that the decreased expression of miR-99b is a candidate biomarker in the prognosis of OS. OS tumor cell proliferation, migration and invasion can be suppressed by the overexpression of miR-99b, indicating the potential of miR-99b as a therapeutic target in the treatment of OS.

## Data Availability

All data generated or analyzed during this study are included in this published article.

## Abbreviations

FZD8: Frizzled-8
KM: Kaplan-Meier
miR-99b: MicroRNA-99b
miRNAs: MicroRNAs
OS: Osteosarcoma
